# Curriculum mapping as a tool to facilitate curriculum development: a new School of Medicine experience

**DOI:** 10.1186/s12909-018-1289-9

**Published:** 2018-08-06

**Authors:** Ghaith Al-Eyd, Francis Achike, Mukesh Agarwal, Hani Atamna, Dhammika N. Atapattu, Lony Castro, John Estrada, Rajunor Ettarh, Sherif Hassan, Shaheen E. Lakhan, Fauzia Nausheen, Tsugio Seki, Matthew Stegeman, Robert Suskind, Anvar Velji, Mohsin Yakub, Alfred Tenore

**Affiliations:** 1 Department of Clinical Sciences, College of Medicine, California Northstate University, 9700 West Taron Drive, Elk Grove, CA 95757 USA; 2 Department of Medical Education, School of Medicine, California University of Science & Medicine, Colton, California USA; 3 Department of Anatomy, Faculty of Medicine, Cairo University, Cairo, Egypt

**Keywords:** Curriculum, Curriculum mapping, Curriculum development, Curriculum management, Accreditation

## Abstract

**Background:**

Every curriculum needs to be reviewed, implemented and evaluated; it must also comply with the regulatory standards. This report demonstrates the value of curriculum mapping (CM), which shows the spatial relationships of a curriculum, in developing and managing an integrated medical curriculum.

**Methods:**

A new medical school developed a clinical presentation driven integrated curriculum that incorporates the active-learning pedagogical practices of many educational institutions worldwide while adhering to the mandated requirements of the accreditation bodies. A centralized CM process was run in parallel as the curriculum was being developed. A searchable database, created after the CM data was uploaded into an electronic curriculum management system, was used to ensure placing, integrating, evaluating and revising the curricular content appropriately.

**Results:**

CM facilitated in a) appraising the content integration, b) identifying gaps and redundancies, c) linking learning outcomes across all educational levels (i.e. session to course to program), c) organizing the teaching schedules, instruction methods, and assessment tools and d) documenting compliance with accreditation standards.

**Conclusions:**

CM is an essential tool to develop, review, improve and refine any integrated curriculum however complex. Our experience, with appropriate modifications, should help other medical schools efficiently manage their curricula and fulfill the accreditation requirements at the same time.

## Background

A curriculum symbolizes the expression of learning and teaching designs in practice [1]. Harden defines the curriculum as “*a sophisticated blend of educational strategies, course content, learning outcomes, educational experiences, assessment, the educational environment and the individual students’ learning style, personal timetable and programme of work*” [2]. A curriculum must be designed such that it is easily communicated to both the students and the faculty, and be effortlessly reviewed, evaluated, and revised once it has been implemented into practice [1].

Curriculum mapping (CM), showing the relationships of all aspects of the curriculum, is a linchpin to attain the objectives/outcomes of any curriculum. It illustrates the relationship between the different components of the curriculum so that all the connections are easily visualized [2]. CM incorporates two singular attributes of any curriculum: communicability and transparency. Transparency and communicability are vital for explaining when, how and what is taught and in what way it is assessed. Unfortunately, both transparency and communicability are often overshadowed by other aspects of the curriculum such as content, pedagogy of engagement, and assessment [2].

Any curriculum, whether well-established or in its developing phase, needs to be continuously monitored, reviewed and evaluated. A well-designed “declared” (or assumed) curriculum may not be a well “delivered” (or taught) curriculum. Likewise, even in a well delivered curriculum, student learning may not be equally well achieved [2, 3]. The reasons behind the discrepancies among the declared (i.e., what the students are expected to learn) curriculum, the delivered (i.e., what is taught) curriculum, and the learned “tested” curriculum may be due to difficulties in the areas of learning expectations (objectives/outcomes), content selection (gaps/unwanted redundancies), content integration, delivery methodology, student learning styles, timing and logistics organization, and assessment strategies. For optimal student learning, the curriculum should be regularly reviewed and updated using an efficient CM process to ensure that what is declared and delivered is in tandem with what is “tested”. Furthermore, accreditation bodies require the curriculum to be transparent enough so that the location of certain learning experiences and their related learning objectives are easily identified. For example, the Liaison Committee on Medical Education (LCME) in the United States requires medical schools to identify the location of objectives for specific topics related to human development such as, adolescent medicine, and geriatrics [4]. Thus, CM becomes the key to making the curriculum transparent and easily communicable and in helping medical schools to achieve compliance with LCME curricular standards.

For several years, the Association of American Medical Colleges (AAMC), which co-sponsors the LCME, has been supporting and encouraging medical schools in the United States to adopt an electronic CM system by offering a Curriculum Management and Information Tool (CurrMIT). This tool helps to manage the curriculum by highlighting its content gaps and redundancies, as well as identifying the role of the faculty in various curricular activities [5]. Currently however, more sophisticated and comprehensive electronic platforms are available from multiple vendors. To further facilitate CM, the AAMC has designed the Curriculum Inventory and Reports (CIR) as a central database for AAMC member medical schools and strongly encourages participation. In this database, information regarding curriculum content and structure, as well as methods of delivery and assessment can be stored. CIR is an important curriculum benchmarking tool that is essential for continuous quality improvement [6]. AAMC and MedBiquitous Curriculum Inventory Working Group have recently published a list of standardized terms (vocabulary) for instruction and assessment [7]. This unified terminology helps the AAMC to aggregate data from different medical schools in order to standardize CM among member medical schools. Therefore, ideally, every medical school must align itself to the AAMC by using the standardized curriculum inventory vocabulary in the CM process. Consequently, a CM process that enhances curriculum management and facilitate its report of the curriculum data to the AAMC was incorporated into curriculum planning.

The aim of this manuscript is to share with other educators how our CM running in parallel with curriculum development effectively guided the curriculum review constantly by (a) identifying content gaps and redundancies; (b) maintaining content integration, and (c) linking learning outcomes across all levels: session, course, program, and Physician Competency Reference Set (PCRS).

## Methods (the mapping process)

**Fig. 1:**
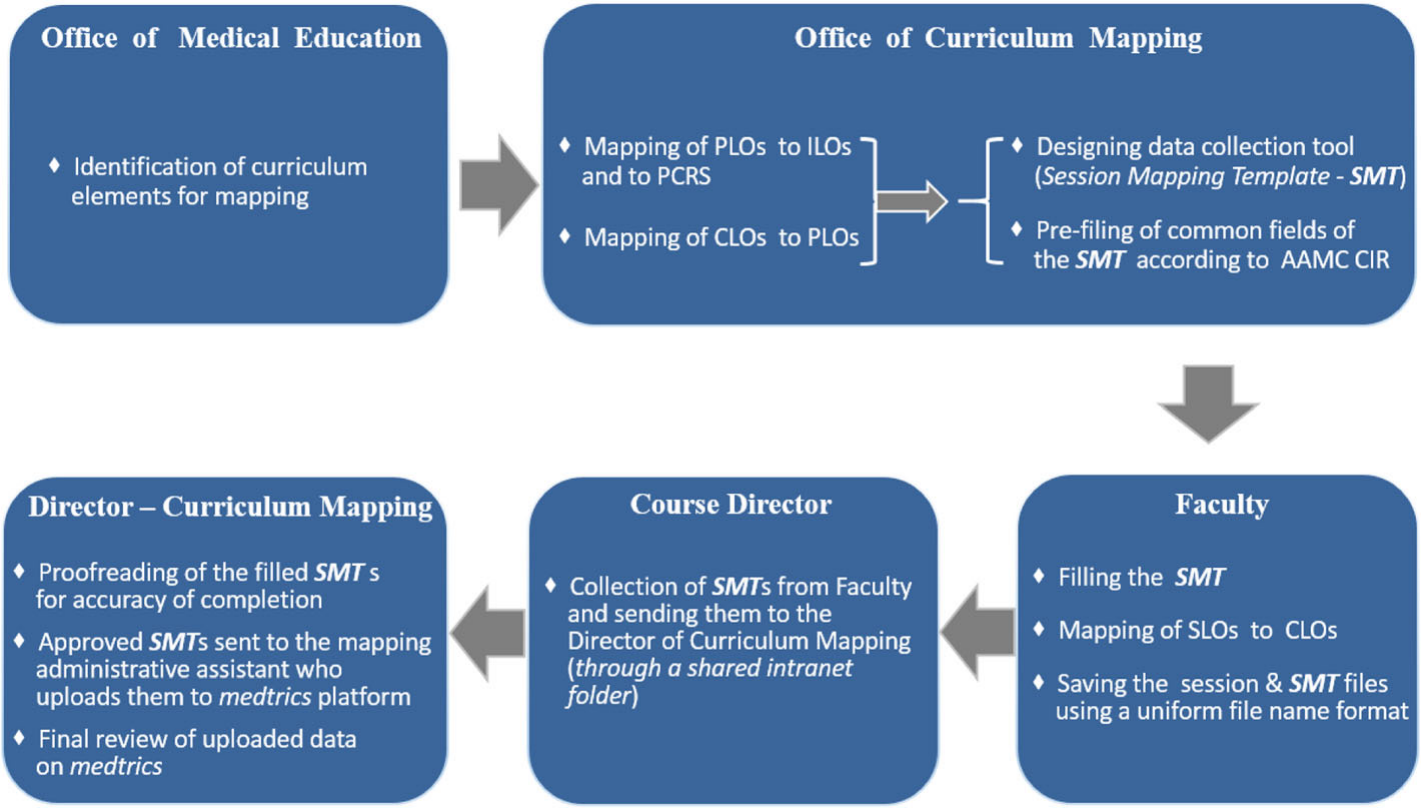
The mapping process

**Fig. 2:**
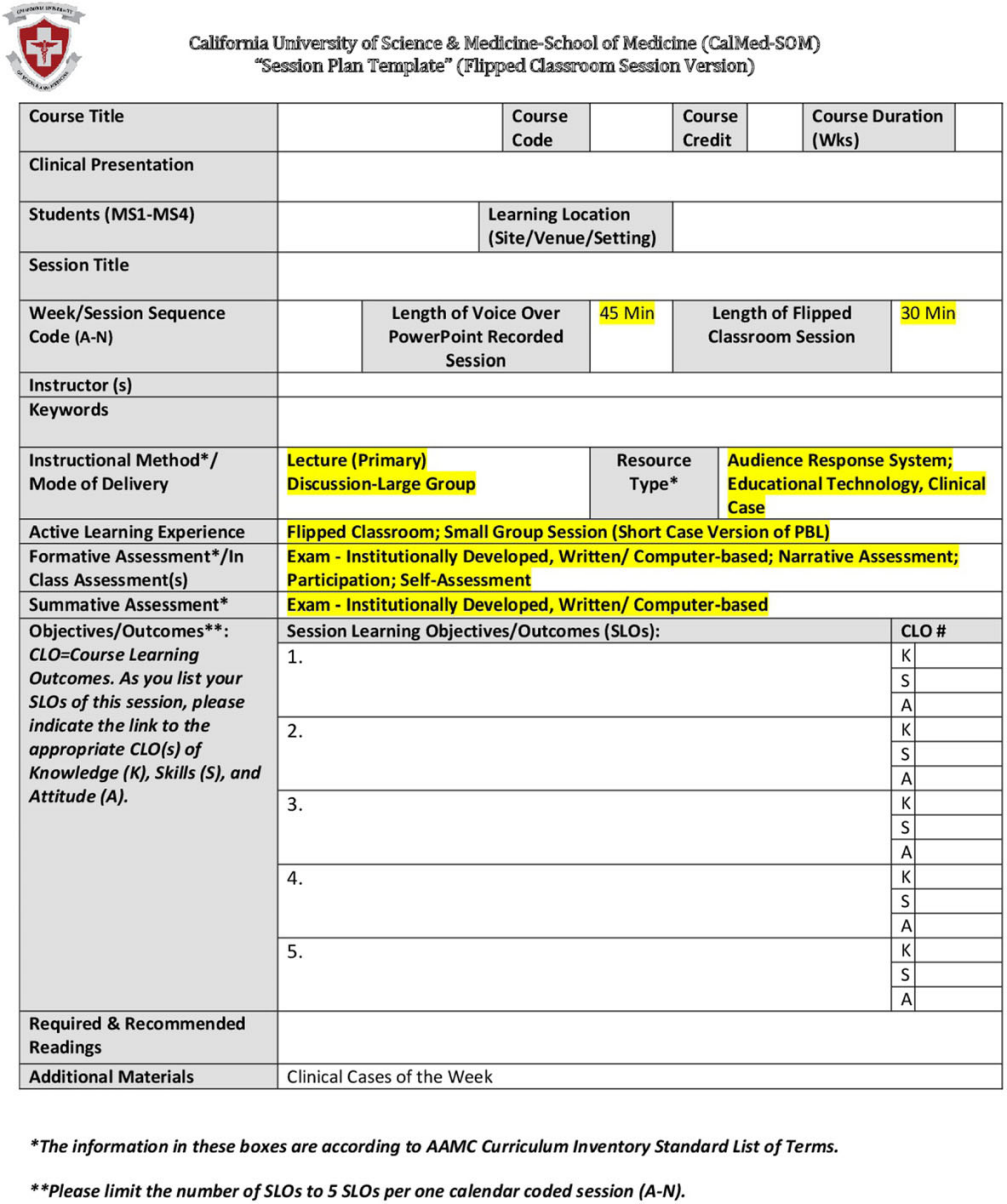
The session mapping template

Our mapping process is summarized in Fig. 1. The Office of Medical Education (OME) identified the elements of the curriculum that need to be mapped under the following four categories: a) “Learning Expectations”, b) “Learning Event Information”, c) “Pedagogy”, and d) “Assessment”. These categories act like windows through which different elements of the curriculum can be viewed and show how different elements of the curriculum (within one category or different categories) are linked [2]. The Learning Expectations category includes the Institutional Learning Outcomes “ILOs”, Program Learning Outcomes (PLOs), Course Learning Outcomes (CLOs), and Session Learning Outcomes (SLOs). The Learning Event Information category incorporates: course code, credit unit, student level, learning venue, session title, session duration, weekly clinical presentation/theme, instructor name, key words/granules, and required reading assignments. The Pedagogy category contains instructional methods and resource type. Lastly, the Assessment category comprises both formative and summative assessment methods. The Office of Curriculum Mapping (OCM) has designed a data collection tool called Session Mapping Template (SMT) that includes a number of fields addressing the key curricular elements mentioned above (Fig. 2). Since all the information regarding schedule, design, teaching methods, resource types, and assessment of the weekly learning sessions of the pre-clerkship curriculum were available in advance and generally uniform, the OCM was able to pre-fill certain fields (highlighted in yellow in Fig. 2) of the SMTs with the existing information, where fields related to instructional methods, resource types, and assessment, were completed with the prescribed terms of the AAMC CIR [6]. The pre-filling of information resulted in development of a specific SMT for each of the following types of educational sessions: a) flipped classroom sessions; b) anatomy lab sessions; c) pathology/histology and other laboratory sessions; d) clinical skills sessions; e) small group sessions; f) college colloquium sessions; g) journal club sessions; and h) service learning/inter-professional education experience sessions (see Fig. 1 for an example of flipped classroom sessions SMT). These templates were made available to all faculty who were required to fill the remaining fields of the template such as session title, session sequence, clinical presentation of the week, students’ academic level, learning venue, faculty name, and session key words/granules. The faculty were also required to map the SLOs of each session to the CLOs and fill the mapping fields of the template accordingly. Initially, the OCM, using excel sheets, had manually mapped PLOs to ILOs, PLOs to PCRS, and CLOs to PLOs. However, after exploring available options a curriculum management system (“medtrics”) [8] was acquired; subsequently, the mapping data of ILOs, PLOs, PCRS, and CLOs were uploaded onto the system, and the mapping templates were built electronically into this system, followed by faculty and staff training.

The mapping template of each session, for all courses, was filled by each faculty member immediately after developing the session’s contents. In addition to other learning events, the pre-clerkship curriculum has a total of seven hours of flipped classroom sessions each week during which, a maximum of fourteen voice-over PowerPoint presentations (45 min each), sequenced from “A” to “N” are discussed. To enhance the filing and retrieval process, a uniform format was adopted for the file names of both the PowerPoint session and its SMT files. This format includes a specific course code, week number, session sequence letter (for flipped classroom sessions), and session title. The course director coordinated the collection of the completed SMTs and forwarded them to the Director of CM who in turn checked all SMTs for completion. Once the SMTs were approved, the Director of CM sent them to the Mapping Administrative Assistant who entered the template data onto the “medtrics” system. Finally, the Director of CM checked the “medtrics” database for accuracy of data entry and made sure that all the information on the template was accurately uploaded into the system.

Building a curriculum map, simultaneously with the development of the curriculum, resulted in the creation of a searchable database available to all faculty. This database was then used to review the curriculum and align its content according to the PLOs and the LCME requirements.

## Results

CM was the principal tool used in the curriculum review process conducted by the Curriculum Committee after each course director reported on their planned course to the Committee. Reports generated from the mapping system were used by the Committee to modify the curriculum and maintain the content integration across both disciplines (horizontal integration) and time (vertical integration) throughout the four years of the curriculum. The mapping system was designed to be used by all parties involved i.e., OME, faculty and students, to obtain information related to learning expectations, learning events, pedagogies, and assessment. In addition, the mapping system was designed to verify the compliance of the curriculum with expected standards (e.g., LCME and other visiting survey teams).

**Fig. 3:**
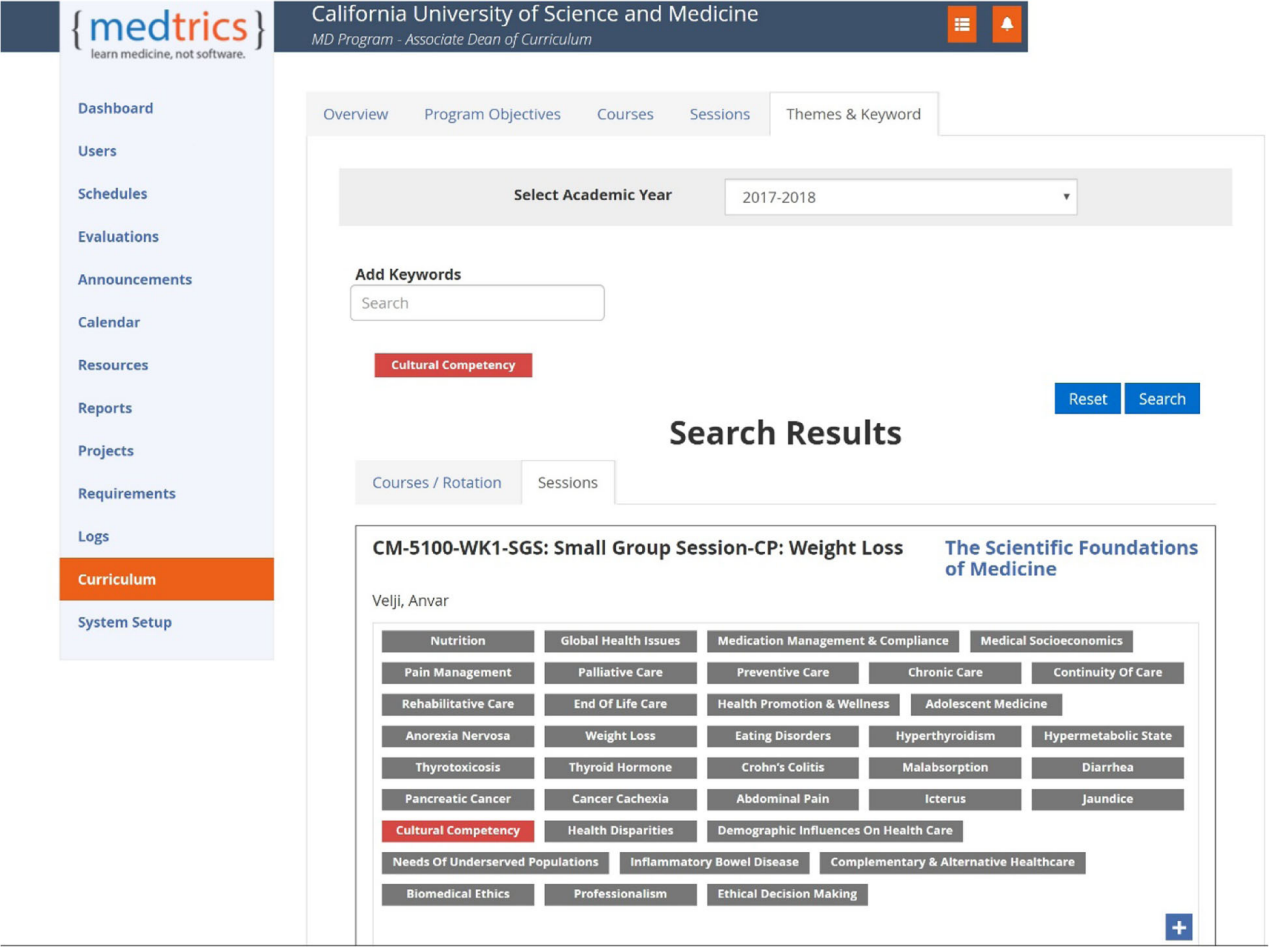
An example of the search results for specific content “cultural competency” in the curriculum database

**Fig. 4:**
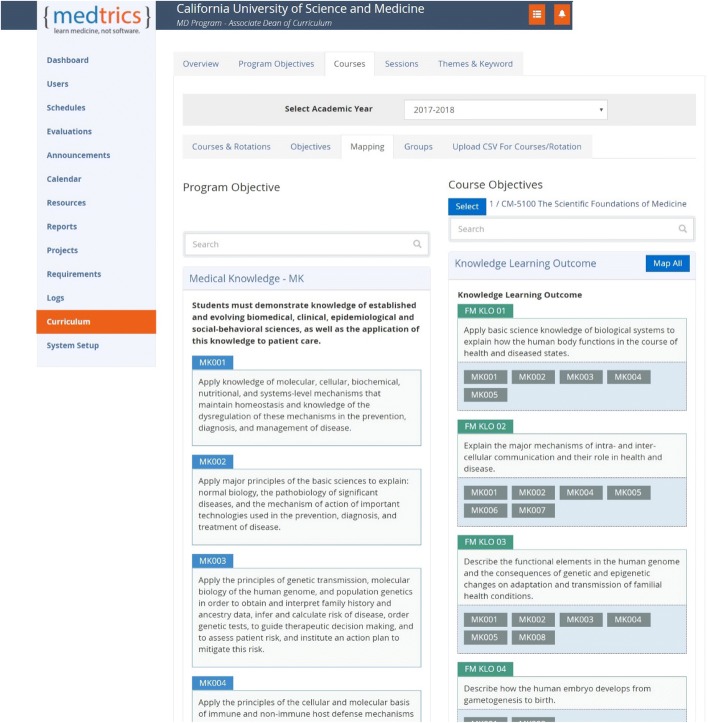
An example of the mapping of CLOs to PLOs

The curriculum map illustrates the curriculum at several different levels as exemplified by Figs. 3 and 4 that indicate examples of keyword search and mapping of CLOs to PLOs. As can be appreciated, the visualization capability of the mapping system was useful in retrieving information related to the links across different levels of learning outcomes and their associated learning events, pedagogy, and assessment. The keyword search capability of the system was useful in reviewing course content for gaps and redundancies as well as in verifying compliance of the course content with certain LCME standards, e.g. Standard-7, and with the topics listed in the USMLE Content Outlines.

The OCM used some of the questions of the LCME Standards for improvement, e.g. a keyword search using the topics listed in LCME elements 7.1 and 7.2 revealed a deficiency in covering “Bioinformatics”. Accordingly, the OME addressed this deficiency and identified the locations within the curriculum where the bioinformatics should be covered. Multiple other gaps and redundancies were identified as shown in Table 1. Many were gross deficiencies which could only be identified by the curriculum map; for instance, the anatomy of the larynx was omitted.

**Table 1:** Deficiencies identified by curriculum review (selected examples)

Course	Gaps	Redundancies
Skin-Musculoskeletal System	Pathology: Tissue Repair and Wound Healing Anatomy: Autonomic Nervous System	Anatomy: Vertebral Canal and Peripheral Nerve
Gastro-Intestinal System	None	Pharmacology: Drug Metabolism and Excretion
Renal System	Pharmacology: Management of Urinary Incontinence	None
Hematology	Pharmacology: Cancer Chemotherapy; Management of Hematolymphoid Malignancies; Management of Bleeding Diatheses & Hypercoaguable Conditions Physiology: Determination of Blood Groups Nutrition: Vitamins Essential for Hemoglobin Synthesis	Biochemistry: Major histocompatibility complex
Cardiovascular-Pulmonary System	Anatomy: Anatomy of the Larynx	None

The results of searching the CM database using keywords related to basic & clinical sciences demonstrated the evidence for the horizontal, vertical, and spiral integration of the curriculum. For example, the CM showed that the basic and clinical sciences were interacting continuously throughout the curriculum, with the spiral integration being evident in the clinical presentation sessions.

The CM system could also be readily used by LCME and other survey teams to identify the locations where certain topics and their learning outcomes were addressed in the curriculum and whether they were adequately addressed.

## Discussion

Medical schools of today are gradually departing from the traditional curricula to more integrated ones. Developing a curriculum in which the basic and clinical sciences are integrated across all four years faces many challenges, which can be overcome using an efficient CM process. An integrated medical curriculum requires that much of the curricular content, traditionally delivered during the clerkship phase of medical school, be introduced in the pre-clerkship phase. Similarly, basic science principles, stressed in pre-clerkship phase, need to be revisited in the clerkship phase.

The careful and balanced blend of curricular content necessary to maintain the integration across all phases of the curriculum requires an efficient CM process that runs simultaneously with curriculum development. This CM process is vital to monitor placing of content, planning of learning events, and aligning assessment with the expected learning outcomes. Our newly designed integrated curriculum incorporates active-learning activities collated from educational institutions worldwide that are guided by adult learning strategies. These learning activities have been refined to focus on some of the shortcomings of current medical education systems which were identified by the Lancet Global Commission on Education of Health Professionals for the twenty-first Century as being fragmented, outdated, locally confined and static [9]. This new curriculum also carefully incorporates the LCME required experiences of self-directed learning as well as principles of patient care across the life cycle (e.g. adolescent medicine, geriatrics, etc.), different levels of patient care, cultural competency, and other experiences stated in LCME Standard-7 [4].

Our curriculum integrates basic and clinical sciences, across disciplines (“horizontal”) within a finite time period, and across time (“vertical”) thereby disrupting the traditional barrier between basic and clinical sciences, as well as across both time and disciplines in the context of common themes throughout all phases of the curriculum (“spiral”) [10].

The challenge of addressing all traditional discipline-based curricula learning expectations in our new integrated curriculum was tackled by incorporating all these objectives under three domains: Knowledge, Skills, and Attitude. The intricate and complex blend of content and pedagogies of this curriculum necessitated using an efficient mapping process that would ensure what is taught is in line with what is assessed and that the curriculum is in compliance with the accreditation standards.

Our experience with CM has shown that a centralized approach of data collection, processing, and uploading to a mapping platform was very efficient in creating a curriculum database that hosts an intricate blend of information. However, other studies have shown that both centralized and decentralized models of CM were both viable approaches that succeeded in creating curriculum map and database for medical curricula [11].

In an outcome-based education such as ours, all decisions related to content selection, educational strategies, educational environment, and teaching and assessment methods are based on how to achieve the desired learning outcomes of the program. Transparency and communicability of the curriculum, especially the learning expectations (objectives/outcomes), to faculty, learners, and curriculum developers, are essential requirements of a successful outcome-based curriculum [12]. Building an efficient curriculum map simultaneously with developing the curriculum ensured that correct linkage occurred between the three levels of session (SLOs), course (CLOs), and program (PLOs) learning outcomes, and that the session content and assessment effectively matched with these outcomes.

An important potential future role of our curriculum map will be the ability to compare the delivered and tested curricula after the curriculum is implemented. Some studies have shown that by analyzing the curriculum map, it is possible to show a discrepancy between the delivered and tested curricula [13, 14]. Other studies have used the curriculum map to reveal curricular elements (e.g. “cultural competency”) which are difficult to recognize in the curriculum because the language of the learning objectives may not be explicit enough, even though the intended learning expectations in those objectives address the topic [15]. Similarly, studies have found CM useful in matching content to outcome, revealing connections between content/disciplines within one course as well as across the whole curriculum, informing both the faculty and learners of content flow, and identifying gaps and redundancies [16, 17].

## Conclusions

The CM process which we used was based on: 1) a mapping template, i.e., SMT, prefilled with standardized data (e.g., AAMC curriculum inventory); 2) a curriculum mapping system (in our case “medtrics”), with well-defined links between learning expectations and curriculum content, and 3) a curriculum review process (course faculty and the Curriculum Committee). CM when simultaneously linked to curriculum development made the curriculum transparent and communicable; it identified content gaps and redundancies and strengthened the content integration. As our medical education program advances, CM will be used to match content to desired outcomes and advance our goal of making medical students to become highly competent and well-trained physicians.

## Abbreviations

AAMC: Association of American Medical Colleges
CIR: Curriculum inventory and reports
CLOs: Course learning outcomes
CM: Curriculum mapping
CurrMIT: Curriculum management and information tool
ILOs: Institutional Learning Outcomes
LCME: Liaison committee on medical education
OCM: Office of Curriculum Mapping
OME: Office of medical education
PCRS: Physician competency reference set
PLOs: Program learning outcomes
SLOs: Session learning outcomes
SMT: Session mapping template
